# Supplementary material for: Exendin-4 Promotes Schwann Cell Survival/Migration and Myelination In Vitro
Source: Int J Mol Sci. 2021 Mar 15;22(6):2971. doi: 10.3390/ijms22062971 (PMC7999558; doi:10.3390/ijms22062971)
Supplement: Supplementary file 1 [file ijms-22-02971-s001.pdf]

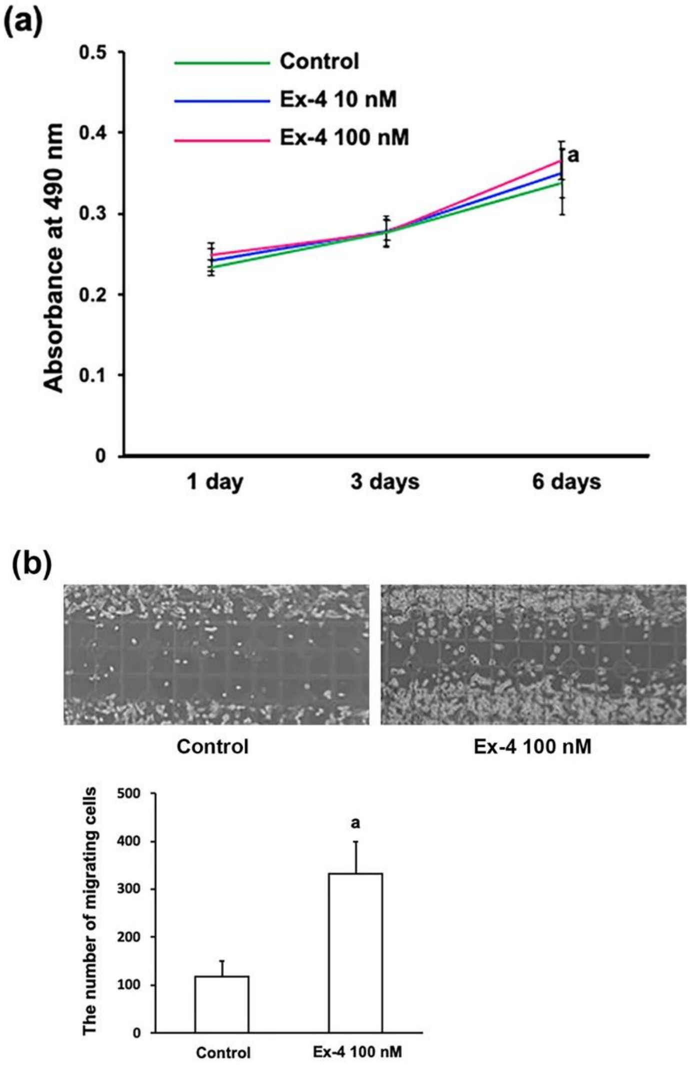

Supplementary Figure 1. Ex-4 promotes survival/proliferation and migration of 1970C3 cells. (**a**) MTS assay; the absorbance at 1 day, 3 days, and 6 days after treatment with 0 (Control), 10 nM and 100 nM Ex-4. Values represent means ± SD from 12 experiments. a: $P<0.05$ as compared with Control. (**b**) Scratch wound assay; representative photomicrographs of 1970C3 cells (**upper**) and the number of migrating cells at 1 day after scratch and treatment with 0 (Control) and 100 nM Ex-4. Values represent means + SD from 9 experiments. a: $P<0.01$ as compared with Control.
